# Clinical Genetics of Prolidase Deficiency: An Updated Review

**DOI:** 10.3390/biology9050108

**Published:** 2020-05-21

**Authors:** Marta Spodenkiewicz, Michel Spodenkiewicz, Maureen Cleary, Marie Massier, Giorgos Fitsialos, Vincent Cottin, Guillaume Jouret, Céline Poirsier, Martine Doco-Fenzy, Anne-Sophie Lèbre

**Affiliations:** 1 Service de génétique, AMH2, CHU Reims, UFR de médecine, 51100 Reims, France; mmassier@chu-reims.fr (M.M.); cpoirsier@chu-reims.fr (C.P.); mdocofenzy@chu-reims.fr (M.D.-F.); 2 SFR CAP SANTE, UFR de médecine, 51100 Reims, France; aslebre@chu-reims.fr; 3 CESM—Pôle de Santé Mentale, CRIA, CIC-EC 1410 CHU de La Réunion, 97448 Saint-Pierre CEDEX, La Réunion, France; michel.spodenkiewicz@inserm.fr; 4 Equipe MOODS Inserm U1178, CESP, 94807 Villejuif, France; 5 Great Ormond Street Hospital NHS Foundation Trust and NIHR Biomedical Research Centre, London WC1N 3JH, UK; maureen.cleary@gosh.nhs.uk; 6 The European Center for Genetics and DNA Identification, DNAlogy. 98 Vouliagmenis Ave. Glyfada, 16674 Athens, Greece; fitsialos@dnalogy.eu; 7 Department of Respiratory Medicine, National Reference Coordinating Center for Rare Pulmonary Diseases, Louis Pradel Hospital, Hospices Civils de Lyon, Lyon, France; Claude Bernard University, Lyon 1, UMR754, IVPC, F-69008 Lyon, France; vincent.cottin@chu-lyon.fr; 8 National Center of Genetics—Laboratoire National de Santé, L-3555 Dudelange, Luxembourg; Guillaume.Jouret@lns.etat.lu; 9 EA3801, 51100 Reims, France; 10 Pôle de Biologie Territoriale, CHU Reims, Service de Génétique, 51100 Reims, France

**Keywords:** prolidase deficiency, *PEPD* gene, systematic review

## Abstract

Prolidase is a ubiquitous enzyme that plays a major role in the metabolism of proline-rich proteins. Prolidase deficiency is a rare autosomal recessive inborn metabolic and multisystemic disease, characterized by a protean association of symptoms, namely intellectual disability, recurrent infections, splenomegaly, skin lesions, auto-immune disorders and cytopenia. To our knowledge, no published review has assembled the different clinical data and research studies over prolidase deficiency. The aim of this study is to summarize the actual state of the art from the descriptions of all the patients with a molecular diagnosis of prolidase deficiency reported to date regarding the clinical, biological, histopathological features, therapeutic options and functional studies.

## 1. Introduction

Prolidase is a ubiquitous cytosolic dipeptidase that liberates proline or hydroxyproline in the final stage of endogenous and dietary protein catabolism. Prolidase contributes to the turnover of collagen and other proline-containing proteins [1,2,3].

Pathogenic variants in the *PEPD* gene (OMIM*613230) encoding prolidase cause a rare recessive inborn error of metabolism named prolidase deficiency (PD) (OMIM#170100) [4,5,6]. PD requires a multisystemic therapeutic approach of each symptom, currently without any definitive cure [7,8,9,10].

Due to a severely reduced prolidase activity in PD, a large amount of proline remains in the form of imidodipeptides X-Proline and X-Hydroxypyroline, which are excreted in the urine [11]. Thus, the hallmark of PD is a massive imidopeptiduria associated with elevated proline or hydroxyproline containing dipeptides in plasma [3,6,11,12,13]. The confirmation of PD diagnosis relies on the measurement of the cellular prolidase activity and on the identification of *PEPD* gene variant [4,12,14,15]. The intra/extra-familial variable expressivity and the lack of correlation between phenotype and genotype are not yet understood [16,17,18].

The incidence of PD is of 1–2 per 1 million births [19,20], but is more frequent in some populations, as the Druze and Arab Muslim minority in Israel [17,18,21]. Since its first description in 1968 by Goodman and colleagues [13], less than a hundred patients with a molecular confirmation for PD diagnosis, from very different ethnic and geographical backgrounds, have been reported [5,18,22].

In this study, we summarize the actual state of the art from the descriptions of all the reported patients with a molecular diagnosis of PD and report a new splicing variant c.1344 + 2T > A in *PEPD*. The aim was, firstly, to describe the phenotypical spectrum of this rare disease with great variability and no agreed therapeutic options and, secondly, report the different functional studies in order to progress in the understanding of this rare disease.

## 2. Materials and Methods

PubMed (https://www.ncbi.nlm.nih.gov/pmc/) and Human Gene Mutation Database Professional (HGMD®, Qiagen, Aarhus C, Denmark) were initially searched up to 1 February 2020. Studies were not excluded based on date of publication. The PubMed search strategy used a combination of medical subject heading (MeSH) terms and text keywords: prolidase and/or *PEPD* gene and prolidase deficiency. This approach was also employed for the other databases, keeping subject headings and keywords as similar as possible between the search strings. We included in this study all the patients reported with a molecular diagnosis of PD. We excluded case reports studies that did not report a genetic analysis. Variant nomenclature were verified with Varsome (Saphetor SA, Lausanne, Switzerland) [23], Mutalyzer (2.0.32) (https://mutalyzer.nl/) [24] and University of California Santa Cruz Genome Browser (http://www.genome.ucsc.edu/) [25]. Prolidase 3D modulization with variant localizations were performed with PyMOL (the PyMOL Molecular Graphics System, Version 1.7, Schrodinger, LLC, New York, NY, USA) and human protein database (5M4Q). DNA sequencing in the reported patient was performed with a BigDye^TM^ Terminator v3.1 cycle sequencing kit on an ABI Prism 3130XL Analyzer (Applied Biosystems, Foster City, CA, USA) following the manufacturer’s instructions. Sequences were analyzed with the SeqScape^TM^ software v.2.5.

## 3. Results

### 3.1. Population

Seventy-five patients have been reported with a molecular analysis of *PEPD,* 34 males and 37 females aged from three months to 47 years (gender data were not available for four patients) (Table S1). Eight patients with PD were known to be deceased between two months and 36 years of age [10,18,22,26,27,28]. Prenatal diagnosis was performed in two families [18,22].

### 3.2. Phenotypical Characterization of Patients with PD

#### 3.2.1. First Symptoms of PD

**Figure 1:**
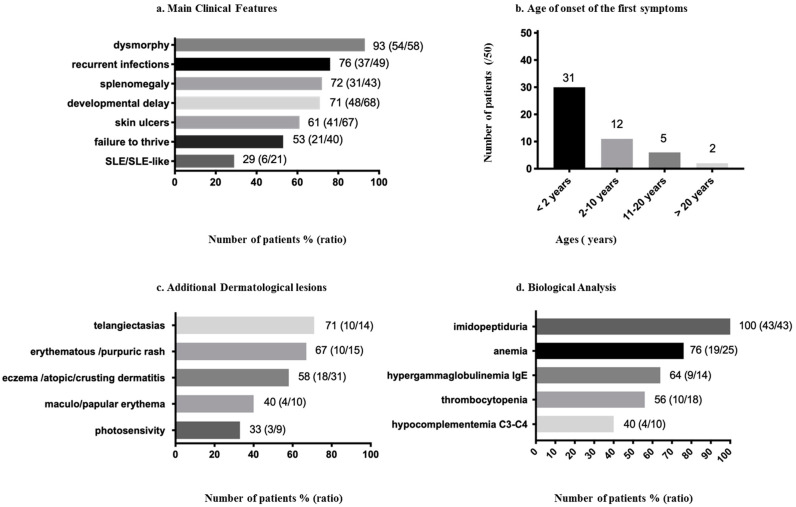
Clinical and biological features reported in prolidase deficiency (PD) patients 1. (**a**) Main clinical features of PD patients. (**b**) Age of onset of the first symptoms. (**c**) Other dermatological lesions. (**d**) Biological analysis.

The first symptoms are an inconstant association of developmental delay, splenomegaly, repetitive infections, dermatological lesions, autoimmune manifestations (“systemic lupus erythematosus (SLE) or SLE-like phenotype” and increased IgE) and cytopenia (anemia and thrombocytopenia) [5,18,26,29] (Figure 1a). Thirty-one patients presented the first symptoms before two years of age (Figure 1b). There is an intrafamilial heterogeneity in the age of onset and severity of symptoms [16,18,22]; two individuals diagnosed with PD were asymptomatic at, respectively, 11 and 29 years of age [16,30]. The dermatological lesions are not necessarily the first signs of the disease, but it is rather an association of symptoms appearing progressively between the neonatal period and adulthood (birth to the third decade) [4,8,17,18,31]. Most patients develop the first symptoms during early childhood, before 10 years of age, but a late onset of leg ulcers appearing during the third decade have also been reported [4] (Figure 1b).

#### 3.2.2. Developmental Delay/Intellectual Disability and Other Neurologic Features

Developmental delay or intellectual disability (moderate, mild or severe) was present in 71% (48/68) patients (Figure 1a). Nevertheless, 20 patients aged from four to 47 were reported without any delay [4,10,16,18,22,28,30,32,33,34,35,36,37], two had normalized their previous developmental delay [16,38], three patients had speech delay [10,18] and one had motor delay with normal intellectual development [16] (Table S1). The expression of developmental delay may vary among siblings [16,35], suggesting that other factors play a role in the severity of the phenotype.

In addition to above, other features noted were bilateral combined deafness, amblyopia, optic atrophy [8] and mixed sensory-neuronal hearing deafness [39]. Seizures were reported in a four-year-old girl who had PD and SLE with central nervous system (CNS) vasculitis. The MRI in this patient showed multiple bilateral subcortical white matter lesions mainly over the parieto-occipital area with leptomeningeal enhancement [29]. Multiple bilateral microthrombosis in the cerebral white matter were found on the MRI in another patient [40]. Computer tomography of the skull of a four-year-old boy with PD showed a slight cortical and cerebellar atrophy [37].

#### 3.2.3. Dysmorphy

Facial dysmorphism was present in 93% (54/58) patients (Figure 1a). Proptosis and/or hypertelorism and saddle nose are reported as a part of the symptoms of PD [18] (Table S1). The majority of patients are described with facial dysmorphism, a peculiar or unusual characteristic [10,18,22,26,29].

#### 3.2.4. Dermatological Symptoms

PD can be associated with a broad scope of dermatological symptoms.

##### Chronic Ulcers

In total, 61% (41/67) of patients were described with cutaneous ulcers (Figure 1a). The cutaneous ulcerations appear in early childhood and may affect children in the first years of life [16,41]. They are chronic, recurrent, extensive, irregular, bilateral, sometimes painful and, especially, predominant on the lower limbs [8,16,31,41,42,43]. It is important to notice that ulcers are often present and suggestive for PD, but their absence does not exclude the diagnosis. The ulcerations may appear on the dorsal part of the foot and on the sole and extend all over the legs, sometimes leading to tendon lesions and severe skin infections [4,8,9,22,26,41]. There were no obvious triggering factors, apart from a trauma described in three patients [16,34,35]. The ulcers may also arise on a previously weakened skin by pruritic or eczematous lesions [8,16,42,44]. Examination of the blood vessels by angiography of the lower extremities in one patient did not show occlusion [8]; venous Doppler examination was reported as normal in another patient [44].

##### Additional Dermatological Signs

Eczema or dermatitis were reported in 58% (18/31) of patients (Figure 1c), described as eczematous skin [16,45] or eczematous lesions on the legs [8] and eczematous eruptions [41]. Crusting dermatitis on the face and extremities was a frequent symptom of PD reported in the Druze population [18].

Telangiectasias were present in 71% (10/14) of patients (Figure 1c), mainly located in the lower limbs but also on the cheeks, shoulders and knees [8,34,35,42,43,46]. A rash was reported in 67% (10/15) of patients (Figure 1c). There is a clinical variability in the presentation of the rash described as a persistent scaling, erythematous with secondary crusts [32], fine purpuric, maculopapular [18,29] or “eczema-like rash” [22]. Another patient had a purple rash localized on the back of his hands and on his earlobes [29]. Photosensitivity was reported in 33% (3/9) of patients (Figure 1c) [4,26].

#### 3.2.5. Recurrent Respiratory Infections and Pulmonary Lesions

Recurrent infections, namely respiratory infections, pneumonia or upper respiratory tract infections [47], are present in 76% (37/49) of patients (Figure 1a). [4,8,10,12,17,21,22,28,31,32,35,38,44,45]. In a retrospective study performed on 21 patients in Israel by Nir et al., 57% of patients, with ages ranging from 10 to 33 years, had a history of recurrent pulmonary infections, and 47% had a diagnosis of chronic lung disease. On the CT scans, different features were found, such as cystic changes, bronchiectasis, diffuse ground glass attenuation and linear atelectasis, suggesting that the respiratory component of the disease should be carefully considered [47]. An additional patient with PD and SLE had pulmonary fibrosis. His videothoracoscopic lung biopsy showed diffuse alveolar fibrosis with excessive collagen deposition, architectural distortion and alveolar cysts [48]. A 16-month-old boy of South Asia with PD was diagnosed with anti-neutrophil cytoplasmic antibody-associated pulmonary capillaritis. Despite apparent good disease control, a CT scan of the chest at the age of five years revealed progressive pulmonary fibrosis and cystic changes. [49].

Recurrent infections, including pneumonia, are a major complication for PD, which can compromise the survival [18,39,47], but no follow-up studies about life expectancy have been published yet.

#### 3.2.6. Failure to Thrive

Fifty-three percent (21/40) of patients presented a failure to thrive (Figure 1a) [18,28,50]. Of these, 12 patients were previously investigated by Besio et al. in the light of bone abnormalities, namely short stature, microcephaly, osteopenia and genu valgum [28]. The features of the skeletal abnormalities were studied thanks to the dal/dal mouse, an animal model for PD that compromised longitudinal bone growth and abnormal geometrical bone properties. This work suggested that lack of prolidase activity is required for normal skeletogenesis, especially at an early age when the requirement for collagen synthesis and degradation is the highest [28].

#### 3.2.7. Gastroenterologic Symptoms

Splenomegaly was found in 72% (31/43) of patients (Figure 1a) [18,22,39], sometimes requiring splenectomy [29]. Hepatomegaly was present in 53% (8/15) of patients [17,22] (Table S1).

An esophagogastroduodenoscopy and colonoscopy performed in a five-weeks-old patient showed scattered gastric and colonic ulcerations [22] and active colitis with multiple linear aphthous ulcers in the left colon in a 21-month-old girl [51]. Colonoscopy in a five-year-old boy showed pancolitis with serpiginous ulcers and pseudopolyps consistent with early Crohn’s disease [50].

### 3.3. Biological Characterization of Patients with PD

#### 3.3.1. Hematologic Disorders

Anemia was reported in 76% (19/25) of patients (Figure 1d) [22,29,31,33,45,52], of which two had SLE [29]. Anemia could be microcytic hypochromic associated with iron deficiency [8,45,53] or hemolytic with a positive Coombs test [21,29]. Thrombocytopenia was found in 56% (10/18) of patients (Figure 1d) [18,22,29,33].

#### 3.3.2. Immunologic Disorders

The immunological disorders included hypergammaglobulinemia with high IgE levels, SLE and hypocomplementemia with low C3-C4 [29,32] (Figure 1d).

Hypergammaglobulinemia IgE was present in 64% (9/14) of patients (Figure 1d) [12,17,22,32,33,53]. The evolution of the IgE levels was reported in one patient. This patient had progressively increasing IgE levels between one and three years of age, reaching 77 600 IU/mL and a Grimbacher score of 34 [32]. Another patient had IgE levels of 1000–2000 IU/mL with a Grimbacher score of 41. A 20-year-old girl had elevated serum immunoglobulin levels of IgE (3300 IU/mL, N = < 100 IU/mL); a comparative expression profile of mRNA involved in the inflammatory response showed increased expressions of IL-23 and TNF-alpha [33].

Systemic lupus erythematosus or SLE-like phenotype was reported in 29% (6/21) of patients (Figure 1a) [16,21,26,29]. Previous studies found that about 10% of PD patients present with complete deficiency have SLE [54]. Several patients, mainly young children, were diagnosed with PD and SLE: a boy aged six years [16], siblings of eight and 12 years [39], and four other unrelated patients of 4, 16, 22 and 24 years [29,48]. Some prolidase-deficient individuals in previous studies only had antibodies against the Sm antigens of the spliceosome, the 60 kD Ro antigen of the Ro-hYRNA complex, chromatin or native DNA, whereas other prolidase-deficient individuals developed an incomplete lupus with serological positivity or a full-blown SLE [54].

Hypocomplementemia was present in 40% (4/10) of patients (Figure 1d) [29,39], of which three had SLE [29]. Serum levels of C1q have been normal [34,54]. CH50 were not reported in the reviewed patients’ cohort. Besides, elevated levels of IgG [8,29,32,39] and decreased neutrophil chemotaxis [32] were also previously described.

#### 3.3.3. Imidopeptiduria

Analysis of urinary amino acids in all tested patients revealed a massive excretion of imidodipeptides such as proline-glycine or proline-hydroxyproline [4,7,11]. Imidopeptiduria is therefore an essential biochemical marker for the diagnosis, the excretion of imidodipeptides being negligible in a healthy person (Figure 1d) [15,19]. The dipeptides also accumulate in the fibroblasts and blood of the patients [4]. Their levels are lower in the serum than in urine [19]. In five patients, the levels of accumulated dipeptides did not correlate with the severity of the disease [4].

Imidopeptiduria is frequently diagnosed by high-performance liquid chromatography (HPLC) analysis by high ninhydrin-positive peaks (Figure 1d). Elevated ninhydrin-positive peaks are secondarily identified after hydrolysis of the urine sample, followed by a second quantitative analysis of the profiles of proline and hydroxyproline. Other diagnostic approaches to detect the urinary imidopeptides are exchange chromatography, thin-layer chromatography, capillary electrophoresis and matrix-assisted laser desorption/ionization time-of-flight mass spectrometry (MALDI-TOF MS) [7,11]. Imidopeptiduria can be detected early during the newborn period, even in an asymptomatic person [4,20]. Nevertheless, increased imidodipeptides excretions have also been reported in cases of patients with increased bone turnover, multiple fractures osteomalacia and rickets [19]. The measurement of the cellular enzymatic activity and/or genetic sequencing of *PEPD* confirm the diagnosis. A study evaluating the levels of urinary proline containing dipeptides did not show any direct levels correlation after supplementations by MnCl_2_, vitamin C and L-proline, although the levels of urinary dipeptides were generally lower during the treatment period [45]. During a trial of apheresis exchanges, repeated monthly for four months, determinations performed on the urine of two patients showed a reduction of imidodipeptides concentrations [55].

## 4. Treatments and Follow-Up

There is neither definitive cure for PD nor consensus for treatment [7,56]. As described below, different approaches were thought to slightly improve the dermatological symptoms in PD: enhancing collagen metabolism with oral supplementation of ascorbic acid or glycine/proline, improving prolidase activity and stability with manganese chlorite [45] and diminishing the immunological reaction by antihistaminic and corticosteroids [29]. Topical application of proline [32,44] or 5% proline and 5% glycine [33] or topical proline application under occlusion were also found to be beneficial in a patient with chronic ulcers [44]. Temporary clinical benefits in the ulcer-healing process were achieved by skin-grafting in one patient [8]. Hyperbaric oxygen therapy, in an attempt to minimize ulcer extension and to decrease the bacterial population, was also performed, with encouraging results [9].

Enzyme replacement therapy has been performed by blood transfusion or allogeneic hematopoietic stem cell transplantation [7,10,53,57]. Erythrocyte transfusion showed a slight improvement in ulcer healing [43,58], as well as apheresis exchanges (replacing prolidase-deficient red blood cells with normal filtered cells) repeated monthly for four consecutive months in two PD patients [55]. Allogeneic hematopoietic stem cell transplantation in one patient showed improvement in prolidase activity; however, the patient died from a secondary infection three months after the transplantation [10]. Due to its invasiveness, other therapeutic approaches have also has been investigated, such as transfusion by previously Mg^2+^-activated erythrocytes [57], adenovirus-mediated gene transfer [59], intracellular delivery of liposome-encapsulated prolidase [60] and pharmacological chaperones [14], which may become future treatments of PD.

For patients with SLE and PD, there are no specific treatment recommendations to date. A girl with SLE, Coombs-positive hemolytic anemia and resistant thrombocytopenia to steroid therapy, cyclophosphamide or intravenous immunoglobulin therapy were reported with an improvement of the hematological and immunological manifestations nine months after a splenectomy [18]. SLE treatment did not show an effect on the skin lesions, as reported in a four-and-a-half-year-old patient who was treated with oral prednisone and hydroxychloroquine [29] and in a 16-year-old girl treated with prednisone, azathioprine and dipyridamole [26]. Rituximab was reported as an effective treatment in lupus nephritis and skin ulcers due to PD in two patients [61].

In the absence of formal surveillance guidelines, an annual check-up is recommended with: skin examination for evidence of malignant transformation in patients with chronic recalcitrant skin ulcers, complete blood count, liver function tests and an abdominal ultrasound examination to assess the sizes of the liver and spleen, as well as a follow-up by a pulmonologist and an immunologist and assessments of motor and cognitive developments [56].

## 5. Prolidase Structure Activity and Regulation

Human prolidase is a glycoprotein that belongs to the pita-bread fold enzymes [6,38,62]. Prolidase cleaves imidodipeptides coming from the intracellular degradation of collagen and other proline-containing proteins, including dietary proteins [2,63]. Its preferential substrate is the glycyl-proline dipeptide [63,64]. Prolidase (also called prolidase I) has an isoform; prolidase II is less characterized but also able to hydrolyze imidodipeptides [65,66]. Contrary to prolidase I, prolidase II shows higher activity with methionine-proline dipeptides [67].

**Figure 2:**
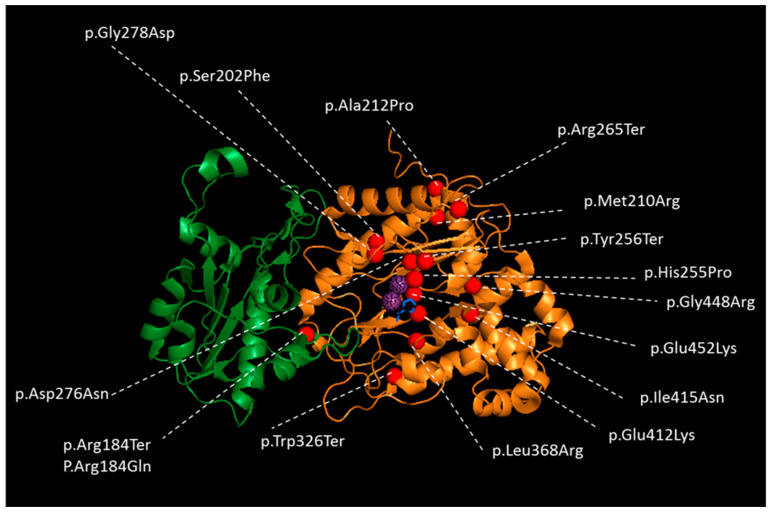
Crystal Structure of one subunit of wild-type Human prolidase dimer as a ribbon representation with reported missense and nonsense variants in patients with PD. The N-terminal domain is colored in green, and the catalytic C-terminal domain in orange. Mn^2+^ ions are represented in dotted, violet spheres and Pro ligand with blue sticks to indicate the location of the active sites of the prolidase dimer. Variants are represented as red spheres. The figure performed using PYMOL (the PyMOL Molecular Graphics System, Version 1.7, Schrodinger, LLC, New York, NY, USA) and human protein database (5M4Q) [70].

Prolidase I is a homodimer composed by monomers of 54.3 kDa each [68,69]. A monomer is formed by an N-terminal domain (Nt, from amino acid number 1 to 184) and a C-terminal catalytic domain (Ct, from amino acid 185 to 493) (Figure 2) [70]. During the maturation of the polypeptide, the Nt chain is processed by removal of the methionine residue and acetylation of the Nt alanine [2,62]. The catalytic domain is created by a “pita-bread” fold and contains a Mn^2+^ center surrounded by two binding pockets. As prolidase also belongs to the family of the metalloproteases, Mn^2+^ is required to stabilize the enzyme and secure the substrate in the binding pockets [1,63,71]. Its optimum catalytic activity is at pH 7.8 and 37–50 °C [72]. The mechanism of the reaction was proposed by analogy with the prolidase of *Escherichia coli and Pyroccocus furiosus* [63,73]. Substrate and product binding were studied on the crystal structure of the wild-type human prolidase [70].

Prolidase activity was severely diminished in all the tested patients ranging from 1–9% of the control, except for one patient with 36% of enzymatic activity in his red blood cells. The latter had recurrent ulcers and intellectual deficiency and died at 11 years of age [28]. The enzymatic activity was tested in the red blood cells, leukocytes, fibroblasts or transfected cells [4,9,10,27,28,72,74]. No correlations between enzyme activities with clinical severity were found [35]. A study of the activity and expression of prolidase in the fibroblast for three different mutants of the *PEPD* gene, p.Glu412Lys, p.Tyr231del and p.Gly448Arg, showed a reduced expression of the protein compared to the wild-type in all the cells, with no correlation between the activity levels and expression suggestive of a compensatory mechanism. In this study, the Vmax in all three mutants was diminished in comparison to the wild-type, and the Km was increased for Gly-Pro and Phe-Pro dipeptides [14].

Animal studies on rats revealed that the activity of the enzyme varies with the developmental stages and the cellular type. Indeed, in rat brains, the activity of prolidase increases three days before birth, reaches a nadir at two days after birth and then gradually increases until day 21 [75]. In the intestinal cells, kinetic parameters (Km and Vmax) were shown to be site-dependent and, thus, different in the duodenum, jejunum and colon. Jejunal and duodenal prolidase were sensitive to dietary restrictions, and their pH activity profiles at 24 h postfeeding were different from that at 48 h postfeeding [76]. Then, also, posttranslational modifications play a role in the regulation of prolidase, namely upregulation of the activity by nitric oxide via the phosphorylation of prolidase serine/threonine residues [77], increase of the manganese concentration [14,37,78] or presence of sulfur amino-acids [66].

## 6. Molecular Genetics

Prolidase is conserved between many species, including archaea and bacteria [1]. The *PEPD* gene encodes prolidase, which contains 493 amino acids [79]. It maps to the chromosome 19q13.11 (GRCh38/hg38), spans 134 kb containing 15 exons (NM_000285.4) and is transcribed into a 2.3-kb mRNA [80].

**Figure 3:**
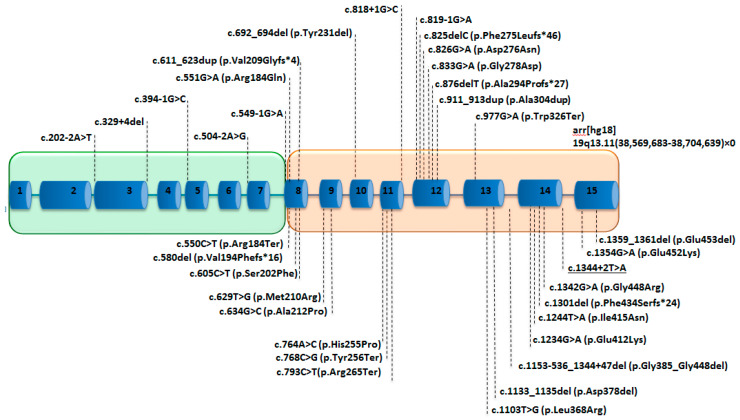
Schematic representation of the *PEPD* with the reported variants in patients with PD. The 15 exons are represented as blue boxes, introns as blue lines. The green box represents the region encoding for the proteic Nt domain, and the orange box represents the region encoding for the Ct domain. The underscored variant is reported for the first time in this study.

Among the 75 PD patients, 35 variants were found, including 16 missense/nonsense variants, 9 splice variants (including the one reported in this paper in our patient), 9 insertions/deletions (indel) and 1 large deletion (copy number variation) (Figure 3) (Tables S1 and S2). The variants are spread along the gene, but most are present within the region encoding for the Ct catalytic domain, especially the missense variants (Figure 3). We observed hot-spot mutations in the 8th, 12th and 14th exons (Figure 3). Five splicing variants concern the Nt-domain, and two variants concern the last codon of the Nt domain; the 27 other variants localized in the Ct domain, and a large deletion encompassed the entire *PEPD* gene [5] (Figure 3). Nonsense variants involving the Ct domain do not predict for a more severe form of the disease [22,28,39,81]. The variants were graded using the American College of Medical Genetics and Genomics variant classification system (Table S2). In this work, we also reported a new splicing pathogenic variant c.1344 + 2T > A in a four-year-old girl presenting with a failure to thrive, hepatosplenomegaly, recurrent infections and imidopeptiduria (Tables S1 and S2). For the pathogenicity prediction, 85 single nucleotide polymorphisms were studied in silico [82], and additional studies of the structural effect of eight single amino acid variants on high-resolution crystal structures of human prolidase highlighted four possible inactivation mechanisms: disruption of the catalytic Mn_2_(OH^−^) center, introduction of chain disorder along with the displacement of important active site residues, rigidification and flexibilization of the active site [83]. To our knowledge, the correlation between the variants type, enzymatic deficiency and clinical signs has not yet been studied [22].

## 7. Histology of Patient’s and Animal Model’s Tissues

The histological analysis mainly focused on the patient’s skin surrounding the ulcers. The reported dermatological lesions were leukocytoclastic vasculitis [10] and nonspecific inflammatory changes [36]. Deposition of amorphous substance was also described as resembling amyloid fibrils in the vicinity of capillaries. In this case were additionally found, within the endothelial cells of the capillaries, round or ovoid structures (1µm in diameter) with or without membranes, with coarse granules of protein or lipid compounds and high electron density [8]. Fragmentation, as well as irregularities in the collagen, were described in the microscope examination but were not revealed by electronic microscopy. Electron microscopy examination showed swelling of the endothelial cells, constriction of the capillary lumens and thickening of the basal lamina [8]. Long-term cultured fibroblasts from PD patients analyzed with light and electron microscopy were rounder and more branched-out than controls with increased cytosolic vacuolization, interruption of the plasma membrane, mitochondrial swelling and cristae modifications. Light microscopy and capillary electrophoresis analysis also showed a significant intracellular accumulation of imidodipeptides in the cell-layer of all the studied patients, and the study of the mitochondrial transmembrane potential performed using JC-1 showed a decreased mitochondrial membrane potential (cellular damage), leading to the assumption that a lack of prolidase activity in the fibroblast may trigger a necrosis-like cellular death [4].

In mice, prolidase was found to be also expressed in the CNS, namely the cerebellum, hippocampus, caudatum, cortex, midbrain and thalamus [84]. A study on the dark-like mice, the animal model for PD with a 4-pb deletion in exon 14, showed an irregular layering of the dendrites above the hippocampal formation compared to the controls, with especially thinner and interrupted pial basal membrane; abnormal cerebellar cortex lobulation and overgrown blood vessels [84]. A further study confirmed that PD affects neuronal maturation during development of a brain cortex area [85]. Focusing on the cerebellar cortex, thinner collagen fibers and disorganized basement membrane below the pial meninx were described in the same animal model, as well as aberrant cortical granule cell proliferation and migration, associated to defects in brain lamination and, in particular, in maturation of Purkinje neurons and the formation of synaptic contacts [85].

An additional study with the dark-like mouse showed a reduced bone growth (femur length) and structural defects, such as bone volume and trabecular thickness, which was associated with impaired chondrocytes proliferation and an increased apoptosis rate in the proliferative zone of the bone, causing a delay of the formation of the second ossification [28]. The dark-like mouse also developed a congenital heart defect that included septal defects and cardiomyocytes hypertrophy [86], but to our knowledge, PD in humans has not yet been described to be associated with hypertrophic cardiomyopathy.

## 8. Pathophysiology of PD

Physiopathology of PD is not clearly understood, as there is marked phenotypic variability among affected individuals [18,22]. The broad symptomatology of PD may be explained by a major role of prolidase in different tissues and cellular functions. The small number of studied patients in this work may probably under or overestimate the prevalence of the clinical and biological signs found in patients, but it is the first performed study that tried to estimate the prevalence of the main PD symptoms in all the reported PD patients with a genetic diagnosis. The protean association, with a variable intrafamilial expressivity of dermatological lesions, developmental delay, splenomegaly, repetitive infections, autoimmune manifestations (“systemic lupus erythematosus (SLE) or SLE-like phenotype” and increased IgE) and cytopenia (anemia and thrombocytopenia) are thus suggestive of PD (Figure 1) [5,18,26,29,56].

First, the lack of proline or increased accumulation of imidodipeptides resulting from PD may perturb the functions of the imidodipeptides-dependent proteins. In fact, prolidase is involved in the recycling of imidodipeptides containing proteins such as collagen [87]. Collagen is a main structural protein of the extracellular matrix in the various connective tissues of the human body, and its dipeptides present a large substrate for prolidase [88,89,90,91]. Studies of fibroblasts cultures from three prolidase-deficient patients showed an increase in the rapidly degraded collagen and a decrease in the proline pool [92]. It was thus hypothesized that a lack of proline may also have an impact on other proline-dependent proteins, such as glutamatergic neurons [90]. In parallel to a lack of proline, the highly increased accumulation of imidodipeptides, as observed in fibroblasts of PD patients, may have an effect on the cellular functions [4].

Secondly, several studies showed a relation between prolidase activity and factors regulating collagen homeostasis, regulation of cell growth, differentiation and migration. Indeed, β1 integrin is a transmembrane-signaling protein of which the activation [2] leads to the stimulation of transcription factors and the expression of many proteins involved in the latter cellular functions [93,94]. β1 integrin receptor signaling was found to upregulate prolidase activity [2,95,96]. Additional studies in human dermal fibroblasts showed that echistatin (desintegrin) downregulated prolidase activity and expression [2,96]. On the contrary, insulin growth factor, a strong inducer of collagen biosynthesis, upregulated prolidase activity [90,97]. In the animal model, PD affected neuronal maturation, proliferation and migration [85], as well as bone growth and structure [28].

Then, PD symptoms reveal an involvement of prolidase regulation in the immune system. The mechanism by which prolidase deficiency may predispose to SLE is unknown. Defects in apoptosis are important in the pathogenesis of SLE, and a lack of free proline, resulting from PD, may impair apoptosis mediated through the proline oxidase pathway [54]. It was suggested that an impaired resolution of neutrophilic inflammation in PD could result in an increased exposure to autoantigenic material in the setting of acute inflammation, where normal tolerogenic signals are absent, resulting in autoimmunity [54]. Studies on TGFβ, a multifunctional cytokine with an active role in autoimmune functions, cancer, fibrotic and cardiovascular diseases, [98,99] found that inhibitors of prolidase activity induced a decreased expression of TGFβ 1 and of its receptor in cultured fibroblasts [100]. Besides, HIF-1α, a transcription factor important for collagen turnover in the process of inflammation, angiogenic signaling, and immune functions [101,102,103,104], was found to be dependent of prolidase expression in colorectal and breast cancer cells [2,105].

**Figure 4:**
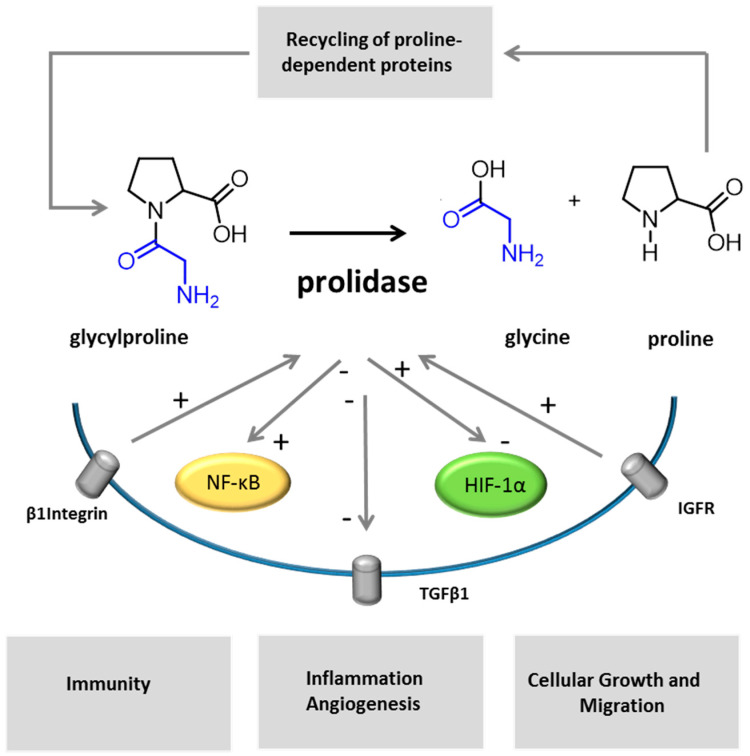
Different mechanisms may be involved in the pathophysiology of PD. Imidodipeptides, as glycylproline, are split out by prolidase. In the representation of the enzymatic reaction, glycine is colored in blue, proline in black. Prolidase activity participates to the recycling of imidopeptide-containing proteins. As reported by previous studies, other cellular factors and receptors are dependent on, or are regulated by, prolidase activity or expression (IGFR, HIF-1α, TGFβ1 and NK-κB). Insulin growth factor and β1 integrin receptor signaling upregulate prolidase activity [2,95,96,97]. Inhibitors of prolidase activity induce a decrease of TGFβ 1 and its receptor [100] and upregulates NF-κB expression [2]. HIF-1α expression was shown to be prolidase-dependent [2,105].

A study also focused on the role of prolidase as a supplier for cells with limited in glucose supplies. Proline may be metabolized into Δ1-pyrroline-5-carboxylate, glutamate and, then, α-cetoglutarate and used for the tricarboxylic acid cycle supply [95] in cancer cells. Regarding cancer, to our knowledge, no patients presenting cancer have been published yet, but this issue may be underestimated in relation to the young cohort of the reported patients. Additional studies reported that the inhibition of prolidase activity upregulated NF-κB expression, an inhibitor of type I collagen gene expression involved in the inflammation, development and regulation of, namely, cytokine, chemokines, cell cycle regulators, adhesion molecules and antiapoptotic factors [2,106,107] (Figure 4).

## 9. Conclusions

PD is a rare but probably underdiagnosed disorder that may escape diagnosis because of its progressive and inconstant symptoms. Since prolidase is a key enzyme for many metabolic and signaling cellular pathways, its deficiency contributes to the development of a large association of dermatological lesions, developmental delay, dysmorphy, splenomegaly, repetitive infections and autoimmune manifestations. Although marked phenotypic variability is not yet understood, this study firstly describes the phenotypic spectrum from all patients with PD and a genetic diagnosis, then reports the histopathological features, therapeutic options and functional studies investigated in PD. This study highlights that a great majority of the variants reported in PD to date are localized in the Ct domain of prolidase. Future whole-genome and multiomics studies in PD patients may help to understand the phenotypic variability among affected individuals, as well as phenotype-genotype correlations, offering some clues and targets for future treatments.

## Supplementary Materials

The following are available online at https://www.mdpi.com/2079-7737/9/5/108/s1, Table S1: Clinico-biological features and genetic results in PD patients. Table S2: Mutations of *PEPD* in PD patients.
